# Clinical, etiological and antimicrobial susceptibility profile of pediatric urinary tract infections in a tertiary care hospital of Nepal

**DOI:** 10.1186/s12887-019-1410-1

**Published:** 2019-01-29

**Authors:** Lok Bahadur Shrestha, Ratna Baral, Prakash Poudel, Basudha Khanal

**Affiliations:** 1 Department of Microbiology and Infectious Diseases, B. P. Koirala Institute of Health Sciences, Dharan, Sunsari 56700 Nepal; 2 Department of Pediatrics and Adolescent Medicine, B. P. Koirala Institute of Health Sciences, Dharan, Sunsari 56700 Nepal

**Keywords:** UTI, Antimicrobial resistance, MDR, MRSA

## Abstract

**Background:**

Urinary tract infection (UTI) is one of most common pediatric infections. The study was designed to assess the clinical profile, common bacterial microorganisms causing UTI and their antimicrobial susceptibility patterns at B. P. Koirala Institute of Health Sciences (BPKIHS) hospital.

**Methods:**

This is a prospective cross-sectional study conducted at Department of Microbiology and Infectious Diseases for 6 months (January to June 2018). A total of 1962 non-repetitive urine specimens (midstream, nappy pad, catheter aspirated) of pediatric patients (0–14 years age) suspected of UTI were obtained in the Microbiology laboratory. Clinical data was obtained from requisition form and hospital software. Culture and bacterial identification was done by using standard microbiological guidelines. Antimicrobial susceptibility testing was done by Kirby-Bauer disc diffusion method following clinical and laboratory standards institute (CLSI) guidelines. Resistance to methicillin and vancomycin were confirmed by calculating minimum inhibitory concentration using broth dilution method.

**Results:**

Among 1962 samples, 314 (16%) were positive for bacterial infection. Fever, irritability and poor feeding was the most common symptoms in neonates while older children presented with fever and urinary symptoms. *E. coli* was reported the most common etiological agent (53%), followed by *Enterococcus faecalis* (22%), *Klebsiella pneumoniae* (7%) and *Staphylococcus aureus* (7%). Multidrug resistant (MDR) isolates accounted for 32% of isolates, while 5% were extensively drug resistant (XDR). Fourty percentage of gram-negative bacilli were ESBL producer, 38% of *S. aureus* were methicillin resistant *Staphylococcus aureus* (MRSA) and 5% *E. faecalis* were vacomycin resistant enterococci (VRE). *E coli* was highly resistant to Ampicillin (87%), Ceftriaxone (62%) and Ofloxacin (62%). Amikacin (11% resistance) and Nitrofurantoin (5% resistance) are the most effective drugs for gram-negative bacilli (GNB) while vancomycin and linezolid are functional against gram-positive cocci.

**Conclusions:**

High-level antimicrobial resistance was observed in pediatric UTI with alarming incidence superbugs like MDR, XDR, ESBL and MRSA. Regular surveillance should be carried out to determine the local prevalence of organisms and antimicrobial susceptibilities in order to guide the proper management of children.

## Background

Urinary tract infection (UTI) are one of the commonest cause of febrile illness in pediatric population with a worldwide prevalence of 2–20% [1, 2]. They can be associated with high morbidity and long-term complications such as renal scarring, hypertension, and chronic renal failure [3, 4]. Pediatric UTI cases remain under-diagnosed in many instances due to absence of specific symptoms and signs, especially in infants and young children [5]. It has been estimated that around 50% of UTI in children are missed [2, 6]. Timely diagnosis and targeted treatment decrease the risk of renal scarring and other complications [7, 8]. For this purpose, empirical antibiotic is often prescribed even before the culture results are available. On the other hand, antibiotic resistance of urinary tract pathogens has been increasing globally [9].

In Nepal, pediatric UTIs are usually treated empirically because of the unavailability of standard therapeutic guidelines and local susceptibility data [10]. In this perspective, the present study was designed to investigate the prevalence, clinical profile, organism spectrum and antimicrobial resistance profile in pediatric UTI in a tertiary care teaching hospital in Nepal.

## Methods

### Study design and setting

This is a cross-sectional study conducted in the Department of Microbiology, B.P. Koirala Institute of Health Sciences (BPKIHS), Dharan, Nepal, for a period of 6 months (1st January-30th June 2018). Patient’s information was collected from requisition form, laboratory records and medical records.

### Laboratory methods

A total of 1962 non-repetitive urine specimens (Midstream clean catch, nappy pad, catheter aspirated) of pediatric patients (0–14 years age) suspected of UTI were obtained in the Microbiology laboratory. To minimize contamination, clean catch midstream method was employed wherever possible. In neonates and early infants, nappy pad method, described by Liaw et al. [11] was used. In case of catheterized patients, urine specimen were collected either through the catheter collection port or through puncture of the tubing with a sterile needle [12]. The samples were then processed by semi-quantitative streaking method using a calibrated inoculating loop (holding 0.001 ml urine) onto the cystine lactose electrolyte deficient (CLED) agar. The inoculated plates were incubated for 24 h at 37 °C in aerobic atmosphere. The isolates were identified using standard microbiological methods that includes colony morphology, gram-stain, catalase, oxidase and an in-house set of biochemical tests [13].

### Antimicrobial susceptibility testing

Antimicrobial susceptibility was tested by modified Kirby-Bauer disc diffusion method on Mueller Hinton agar (Hi-Media, India) following standard procedures recommended by the Clinical and Laboratory Standards Institute (CLSI) [14]. Antibiotics that were tested in our study include: ampicillin (10 μg), amoxicillin clavulanate (20/10 μg), amikacin (10 μg), high level gentamicin (120 μg), co-trimoxazole (1.25/23.75), cephalexin (30 μg), ceftriaxone (30 μg), ceftazidime (30 μg), cefotaxime (30 μg), colistin (10 μg), ofloxacin (5 μg), piparacillin (100 μg), piperacillin tazobactam (100/10 μg), imipenem (10 μg), penicillin G (10 units), vancomycin (30 μg), linezolid (30 μg). Interpretations of antibiotic susceptibility results were made according to the zone size interpretative standards of CLSI. *Escherichia coli* ATCC 25922 and *Staphylococcus aureus* 25923 were used as a control organism for antibiotic susceptibility testing [14]. Resistance to methicillin and vancomycin in *S. aureus* and vancomycin resistant enterococci were confirmed by calculating the MIC of the antibiotics using broth dilution method [15].

### Identification of multidrug resistant (MDR) and extensive drug resistant (XDR) organisms

The isolates were identified as MDR and XDR on the basis of combined guidelines of the European Centre for Disease Prevention and Control (ECDC) and the Centers for Disease Control and Prevention (CDC) [16].

### Screening and confirmation for ESBL production

Gram-negative bacilli were screened for ESBL production by using third generation cephalosporins discs i.e. ceftazidime (30 μg), cefotaxime (30 μg) and cefotriaxone (30 μg). If the zone of inhibition (ZOI) was ≤25 mm for ceftriaxone, ≤22 mm for ceftazidime and/or ≤ 27 mm for cefotaxime, the isolate was considered a potential ESBL producer and confirmed by Combination disc test (CDT) method. In this method, the organism was tested against ceftazidime (30 μg) disc alone and ceftazidime+ clavulanic acid (30/10 μg) combination disc. Isolate that showed increase of ≥5 mm in the ZOI of the combination discs in comparison to that of the ceftazidime disk alone was considered an ESBL producer [14].

## Results

During the study period (1st January 2018-30th June 2018), a total 1962 urine samples from children with suspected UTI were obtained among which 314 samples (16%) yielded significant bacteriuria. Among 314 positive samples, 168 (54%) were male and 146 (46%) were females. The positivity rate of UTI from clean catch, nappy pad and catheter aspirated urine were 16% (272/1712), 14% (28/200) and 28% (14/50) respectively. The prevalence rates of febrile UTIs in neonates, infants, pre-school and children was 18.6% (28/150), 19% (88/462), 14.9% (80/534) and 14.4% (118/816) respectively. Fever was the most common clinical presentation followed by dysuria and urgency [Table 1]. Among neonates, fever (87%), poor feeding (75%) and irritability (75%) were the most common clinical features.

**Table 1:** Clinical presentation according to age category

	Neonate (*n* = 24)		Infant (*n* = 74)		Pre-school (*n* = 80)		Children (*n* = 136)		Total (*n* = 314)	
	*n*	%	*n*	%	*n*	%	*n*	%	*n*	%
Fever	21	87%	64	86%	70	87%	110	80%	265	84%
Dysuria	–	–	35	47%	50	62%	85	62%	170	54%
Frequency	–	–	30	40%	52	65%	72	52%	154	49%
Urgency	–	–	40	54%	50	62%	74	54%	164	52%
Abdominal pain	–	–	40	54%	45	56%	65	47%	150	47%
Vomiting	8	33%	34	45%	40	50%	40	30%	122	38%
Poor feeding	18	75%	60	81%	30	37%	20	14%	128	40%
Irritability	18	75%	62	83%	25	31%	30	22%	135	42%

**Fig. 1:**
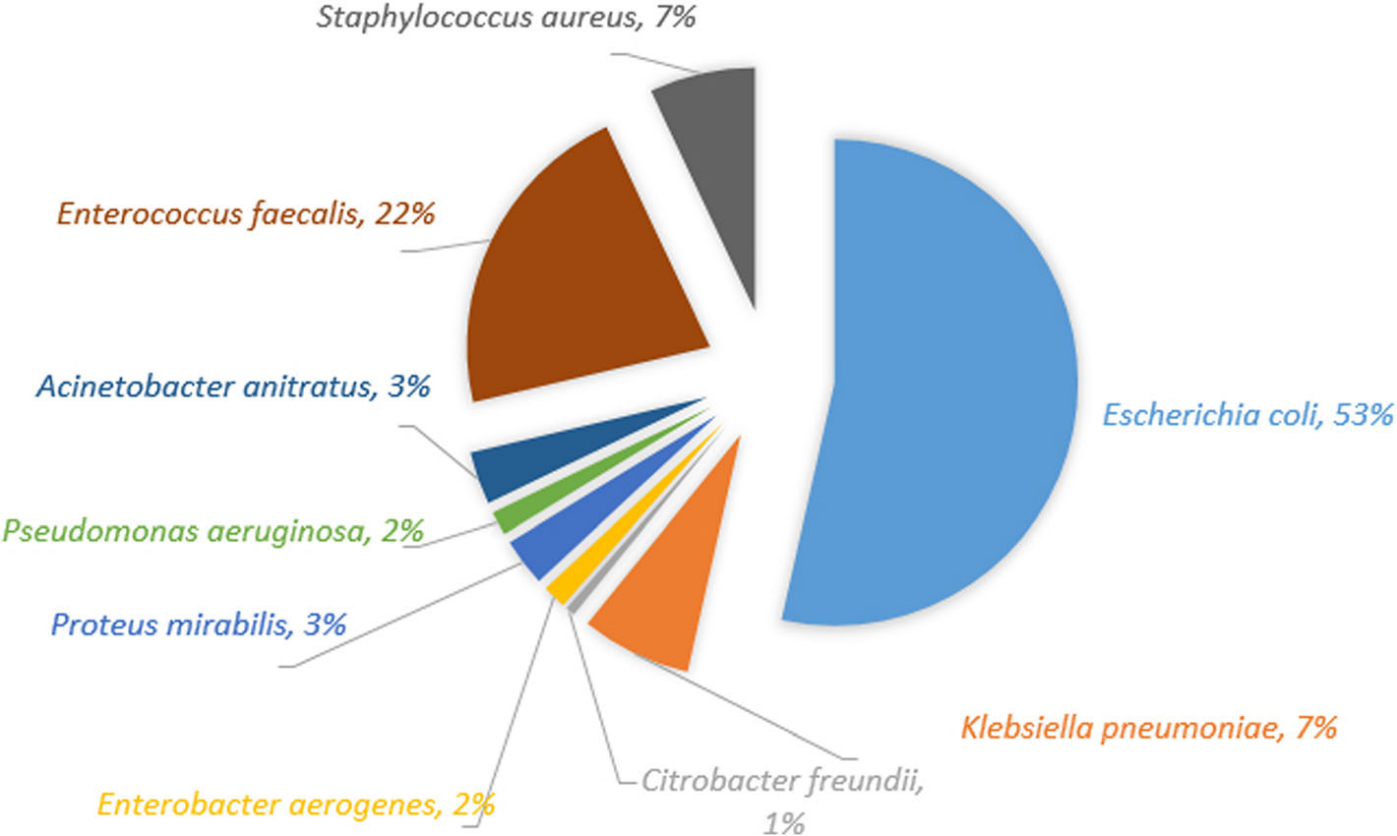
Organism profile

*Escherichia coli* (*n* = 168, 53%) was the most common organism followed by *Enterococcus faecalis* (*n* = 68, 22%) and *Klebsiella pneumonia* (*n* = 23, 7%). The details of organism profile is elicitated in Fig. 1. The organism profile on the basis of age category has been detailed in Table 2.

**Table 2:** Distribution and frequency of uro-pathogens according to age category

Uro-pathogens	Frequency among age-group								Total (*n* = 314)	
	Neonate (*n* = 24)		Infant (*n* = 74)		Pre-school (*n* = 80)		Children (*n* = 136)			
	*n*	%	*n*	%	*n*	%	*n*	%	*n*	%
*E. coli*	12	50%	35	47%	53	66%	71	52%	168	53%
*K. pneumoniae*	5	21%	7	10%	–	–	20	15%	23	7%
*C. freundii*	–	–	–	–	–	–	–	–	2	1%
*E. aerogenes*	1	4%	1	1%	–	–	5	4%	5	2%
*P. mirabilis*	–	–	–	–	4	5%	5	4%	10	3%
*P. aeruginosa*	–	–	–	–	–	–	2	1%	5	2%
*A. anitratus*	1	4%	2	3%	3	4%	2	1%	8	3%
*S. aureus*	2	8%	4	5%	5	6%	12	9%	22	7%
*E. faecalis*	3	13%	25	34%	315	19%	14	10%	68	22%

**Fig. 2:**
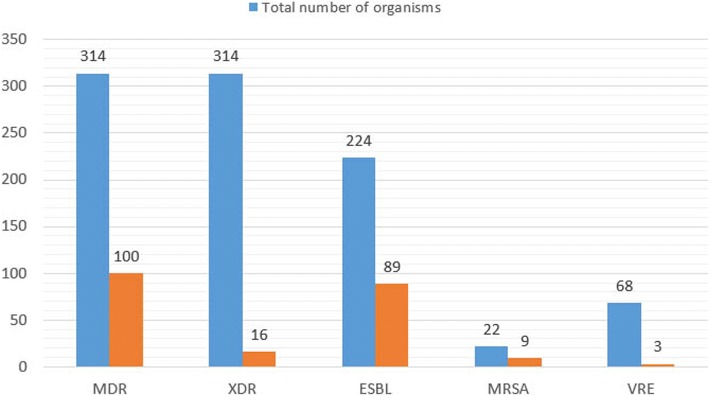
Multi-drug resistant organisms

Antimicrobial susceptibility test showed variable degree of resistance [Table 3]. Eighty-seven percentage of *E. coli* were resistant to ampicillin, 62% to ceftriaxone and ofloxacin. Regarding gram-positive bacteria, 95% of *S. aureus* were resistant to penicillin, 60% to cephalexin and 54% to co-trimoxazole. MDR isolates accounted for 32% (*n* = 100) of the 314 isolates, while 5% (*n* = 16) of them were XDR. Fourty percentage of gram-negative bacilli were ESBL producers. Thirty-eight percentage of *S. aureus* were methicillin resistant *Staphylococcus aureus* (MRSA), while none of them were resistant to vancomycin. Among *E. faecalis*, 5% (*n* = 5) of them were VRE (Fig. 2).

**Table 3:** Antimicrobial resistance pattern of the isolates (resistance in %)

Microorganism	Antimicrobial agents																
	Amikacin	AMC	Ampicillin	Cephalexin	Ceftriaxone	Cefoxitin	Ofloxacin	Nitrofurantoin	HLG	Imipenem	Piperacillin	PIT	Colistin	Cotrimoxazole	Penicillin	Vancomycin	Linezolid
*E. coli*	11	–	87	–	62	–	62	5	–	15	71	14	0	54	–	–	–
*K. pneumoniae*	13	100	–	–	41	–	20	11	–	14	58	20	0	40	–	–	–
*C. freundii*	0	50	50	–	50	–	0	0	–		50	0	0	–	–	–	–
*E. aerogenes*	0	100	100	–	50	–	0	0	–	0	67	0	0	–	–	–	–
*P. mirabilis*	0	100	75	–	0	–	22	75	–	0	0	0	0	–	–	–	–
*P. aeruginosa*	0	–	–	–	67	–	50	80	–	0	50	0	0	–	–	–	–
*A. anitratus*	22		33	–	62	–	22	75	–	14	33	0	0	–	–	–	–
*S. aureus*	21	–	–	60	38	38	42	0	–	–	–	–	–	54	95	0	0
*E. faecalis*	93	–	–	–	–	–	68	10	40	–	–	–	–	–	69	5	0

[−: not tested]

Multi drug resistant isolates were studied on the basis of the type of sample. MDR was seen in 71.4% isolates from catheter-aspirated urine, while only 30.4% isolates from clean catch urine and 28.5% isolates obtained from nappy pad method were MDR (Table 4).

**Table 4:** Multi-drug resistant isolates with respect to the type of samples

	Clean catch	Nappy pad	Catheter aspirated
	Total samples =1712	Total samples = 200	Total samples = 50
	Growth = 272	Growth = 28	Growth = 14
MDR	30.14% (*n* = 82)	28.5% (*n* = 8)	71.4% (*n* = 10)
XDR	3.6% (*n* = 10)	7.1% (*n* = 2)	28.5% (*n* = 4)
ESBL	40% (*n* = 78 of 195 GNB)	40.9% (*n* = 9 of 22 GNB)	28.5% (*n* = 2 of 7 GNB)
MRSA	37.5% (*n* = 3 of 8 *S. aureus*)	33.3% (*n* = 2 of 6 *S. aureus*)	50% (*n* = 2 of 4 *S. aureus*)
VRE	3% (*n* = 2 of 65 *E. faecalis*)	0	33.3 (*n* = 1 of 3 *E. faecalis*)

## Discussion

UTI is a common health problem in children and it is an important cause of morbidity and mortality, especially in the first 2 years of life [17]. In our study, 16% of total samples were positive for UTI. The finding is similar to studies done by Parajuli et al. [18] in Kathmandu, Nepal and Kaur N et al. [19] in India. However, study done by Badhan et al. [20] in India showed a higher (26.7%) culture positivity and some studies showed very low rate of UTI among children i.e. 7.87% in Iran and 9% in USA [6, 9]. UTI is one of a common bacterial infection in children in the world [21].

Children with UTI usually present with non-classical clinical features and these are difficult to diagnose [22]. In our study, fever, poor feeding and irritability were the common clinical features in neonates while the older children presented with fever and urinary symptoms. Our data agree with other reports, where fever, abdominal pain, vomiting, dysuria, poor feeding, and irritability are reported as frequent signs and symptoms of UTIs [23, 24]. Diagnosis of UTI is really challenging due to its vague presenting symptoms, especially in young children. Thus, a high index of suspicion is appropriate when a young child presents with fever [22].

The most common organism associated with Pediatric UTI was *E. coli* (53%). The finding of our study is consistent with many studies [18, 20, 25, 26]. *E. coli* is the most common etiological agent responsible for UTI irrespective of age, sex, community or country and accounts for 50–90% of cases. Uropathogenic *E. coli* (UPEC) originate from the faecal flora, spread across the perineum, and invade the bladder through the urethral opening [20, 22]. In this study, *E. faecalis* comprised of 22% of causative agent and *S. aureus* 7%. Other studies have concluded similar results [19, 27, 28]. Although gram-negative bacteria is responsible for majority of UTI, gram-positive organisms have become important cause of UTI in recent years [29].

The most striking finding of our study is the alarming prevalence of multi drug resistance organisms. Thirty-two percentage of organisms were MDR and 5% were XDR. The finding is similar to study done by Baral et al. [28] and Parajuli et al. [18] in Kathmandu, Nepal. A very high rate of MDR (76.5%) has been reported in India [30]. Among gram-negative bacilli, 40% were ESBL producers. Similar results were reported by Akram et al. (42%) [31], Taneja et al. (36.5%) [32], Parajuli et al. (38.9%) [18] and Fatima et al. (33.5%) [33]. Higher rates of ESBL producers have been reported in other studies [28, 34]. However Wu et al. [35] reported very low prevalence of ESBL producer (14%) in pediatric UTI. Pediatric UTIs due to ESBL-producing bacteria are an important part of the problem as they limit therapeutic choices and increases morbidity of infection [35]. Eighty-seven percentage of *E. coli* were resistant to Ampicillin, 62% to Ceftriaxone and ofloxacin, 54% to cotrimoxazole. The finding is similar to other studies [6, 9, 28]. Our study shows that nitrofurantoin is still the most effective antimicrobial agent for the treatment of UTI. The finding is in agreement with studies done elsewhere [26, 36–38]. .Nitrofurantoin remains a reliable first-line agent for the empirical treatment of acute uncomplicated cystitis [39].

Among gram-positive bacteria, 38% of *S. aureus* were MRSA; 95% of were resistant to penicillin, 60% to cephalexin and 54% to cotrimoxazole. A study conducted in Ireland concluded that 27.8% of *S. aureus* isolated from urine samples were MRSA [40]. Recent studies have reported the increasing prevalence of multi drug resistant *S. aureus especially MRSA* in UTIs [40, 41]. Among *E. faecalis,* 95% were resistant to amikacin, 69% to penicillin and 68% towards ofloxacin. Five percentage were resistant to vancomycin (VRE). All the isolates were susceptible to vancomycin and linezolid. The finding is similar to study done by Kaur et al. [19] in India.

MDR, XDR and MRSA and VRE were noted in higher numbers in case of catheter aspirated urine as compared to clean catch and nappy pad method. Several studies have suggested that isolates obtained from catheterized patient are highly resistant [42, 43]. Previous hospitalization, long-term broad spectrum antimicrobial therapy, co-morbidity, frequent instrumentation, cross transmission of pathogens in catheterized patients might explain the higher antimicrobial resistance [44].

## Conclusion

High-level antimicrobial resistance was observed in pediatric UTI with alarming incidence superbugs like MDR, XDR, ESBL and MRSA. Regular surveillance should be carried out to determine the local prevalence of organisms and antimicrobial susceptibilities in order to guide the proper management of children.

## Abbreviations

ESBL: Extended spectrum β-lactamase
MDR: Multi drug resistant
MRSA: Methicillin Resistant *Staphylococcus aureus*
TDR: Total drug resistant
UTI: Urinary tract infection
XDR: Extensively drug resistant
